# Hepatolithiasis Associated With Anomalous Biliary Anatomy and a Vascular Compression Hepatolithiasis and Anomalous Anatomy

**DOI:** 10.1155/1991/23701

**Published:** 1991

**Authors:** Graham Cullingford, Brian Davidson, James Dooley, Nagy Habib

**Affiliations:** 1 Hepatobiliary and Liver Transplantation Unit Royal Free Hospital and School of Medicine London UK; 2 Department of Surgery Royal Free Hospital Pond Street London NW3 2QG UK

## Abstract

Biliary tract abnormalities occur in about one of every three people, usually being minor and of no clinical significance.Major abnormalities, however, may present in an unusual manner and provide a major hazard to the unsuspecting surgeon.

A patient presenting with cholangitis without jaundice or abnormal liver function tests is reported. Endoscopic retrograde cholangiography failed to demonstrate any bile ducts in the right postero-lateral segments of the liver, the “naked segment sign”. A percutaneous transhepatic cholangiogram demonstrated a stricture obstructing the right posterior segmental hepatic duct with hepatolithiasis above the stricture. At operation an anomalous vessel was found at the site of the stricture.

This case highlights the unusual way in which biliary tract anomalies may present and the importance of adequate pre-operative investigation.


*HPB Surgery*, 1991, Vol. 3, pp. 129–137
Reprints available directly from the publisher
Photocopying permitted by license only

© 1991 Harwood Academic Publishers GmbH
Printed in the United Kingdom

### CASE REPORT

### HEPATOLITHIASIS ASSOCIATED WITH ANOMALOUS BILIARY ANATOMY AND A VASCULAR COMPRESSION

### HEPATOLITHIASIS AND ANOMALOUS ANATOMY

GRAHAM CULLINGFORD, BRIAN DAVIDSON, JAMES DOOLEY and NAGY HABIB

*Hepatobiliary and Liver Transplantation Unit, Royal Free Hospital and School of Medicine, London.*

*(Received 13th June 1990)*

KEY WORDS: Biliary anomalies, vascular anomalies, cholangitis, hepatolithiasis, endoscopic retrograde cholangiography, percutaneous transhepatic cholangiography

Biliary tract abnormalities occur in about one of every three people, usually being minor and of no clinical significance[1]. Major abnormalities, however, may present in an unusual manner and provide a major hazard to the unsuspecting surgeon.

A patient presenting with cholangitis without jaundice or abnormal liver function tests is reported. Endoscopic retrograde cholangiography failed to demonstrate any bile ducts in the right postero-lateral segments of the liver, the "naked segment sign". A percutaneous transhepatic cholangiogram demonstrated a stricture obstructing the right posterior segmental hepatic duct with hepatolithiasis above the stricture. At operation an anomalous vessel was found at the site of the stricture.

This case highlights the unusual way in which biliary tract anomalies may present and the importance of adequate pre-operative investigation.

### CASE REPORT

A 66 year old lady was admitted as an emergency with right sided abdominal pain, weakness, nausea, vomiting and rigors. For the three months preceding this admission she had had occasional central abdominal pains, had felt weak and nauseated and had lost 10 kg in weight. Her past medical history included recurrent urinary tract infections, an appendicectomy and a right mastectomy for carcinoma in 1985.

On examination she was sweaty with a temperature of 38°C, pulse rate of 90/minute and a systolic blood pressure of 100mmHg. She was neither jaundiced nor

Address for correspondence: Mr Brian R Davidson, Department of Surgery, Royal Free Hospital, Pond Street, London NW3 2QG

cyanosed. Abdominal examination revealed diffuse right sided tenderness without rebound or guarding. There was no evidence of recurrence of her breast cancer.

Blood tests showed a haemoglobin of 11.8g/dl (11.5–15.5), white cell count 26.7 $\times$ $10^9$/l (3.8–11.0), normal electrolytes, urea 11.6mmol/l (3.0–6.5), creatinine 117 umol/l (60–120), albumin 31g/l (30–50), bilirubin 8umol/l (5–17), AST 14 IU/l (5–40) and alkaline phosphatase 186 IU/l (35–130). A provisional diagnosis was made of septicaemia with an origin in the biliary tree or urinary tract. Subsequent urine microscopy showed the presence of 200 white blood cells per high power field with no organisms. Blood cultures grew Eschericia Coli.

Abdominal ultrasound examination revealed dilated intrahepatic bile ducts with the appearance of a stone in the cystic duct. The gallbladder and right kidney were normal. Endoscopic cholangiography failed to confirm dilated intrahepatic ducts but showed poor filling of the left hepatic ducts (Figure 1). A nasobiliary drain failed to settle her sepsis and a percutaneous transhepatic cholangiogram was therefore performed, under ultrasound guidance, which revealed dilated right hepatic ducts with several calculi lying above a distal stricture (Figure 2). Infected bile which subsequently grew E. Coli was removed from the dilated segment by external biliary drainage which produced a dramatic reduction in the patients toxicity and fever.

Subsequent comparison of the PTC to the ERCP radiographs revealed anomalous biliary anatomy with the right posterior segmental hepatic duct (RPSHD) inserting into the common hepatic duct (CHD) three centimetres below the confluence of the left hepatic duct and right anterior segmental hepatic duct, which had been previously mistaken for the right hepatic duct. There was complete obstruction of the RPSHD at its insertion into the CHD, just above the cystic duct (Figure 1), with calculi above this obstruction. The nature of this obstruction could not be determined from the cholangiograms.

Despite initial improvement following external biliary drainage she again became septic and was therefore taken to theatre. At laparotomy a dilated RPSHD containing stones was confirmed with a fibrous stricture 3mm proximal to the CHD (Figure 3). At the site of stricture a branch of the cystic artery was passing over the aberrant duct. The duct was explored and several brown pigment stones, extending proximally within the liver substance, were removed. The anomalous duct was divided with closure distally and proximal drainage in a jejunal Roux-en-Y loop.

No malignancy was found in the biopsies submitted for histology. She made a smooth post-operative recovery until the third day when she vomited and inhaled necessitating admission to the intensive care unit for ventilation. She recovered from this episode and has suffered no subsequent attacks of cholangitis. On discharge from hospital her liver function tests were normal.

### DISCUSSION

Drainage of a right posterior segmental hepatic duct into the distal common hepatic duct which has formed by the junction of the left hepatic and right anterior segmental hepatic duct is reported as occurring in 4–6% of individuals[1,2]. This anatomical variation is distinct from the presence of an accessory hepatic duct, such as the subvescical duct, which usually lies in the gallbladder fossa and drains into the right hepatic duct. This duct, which may occur in 20–35% of people[2,3], drains only a small area in the right posterior segment.

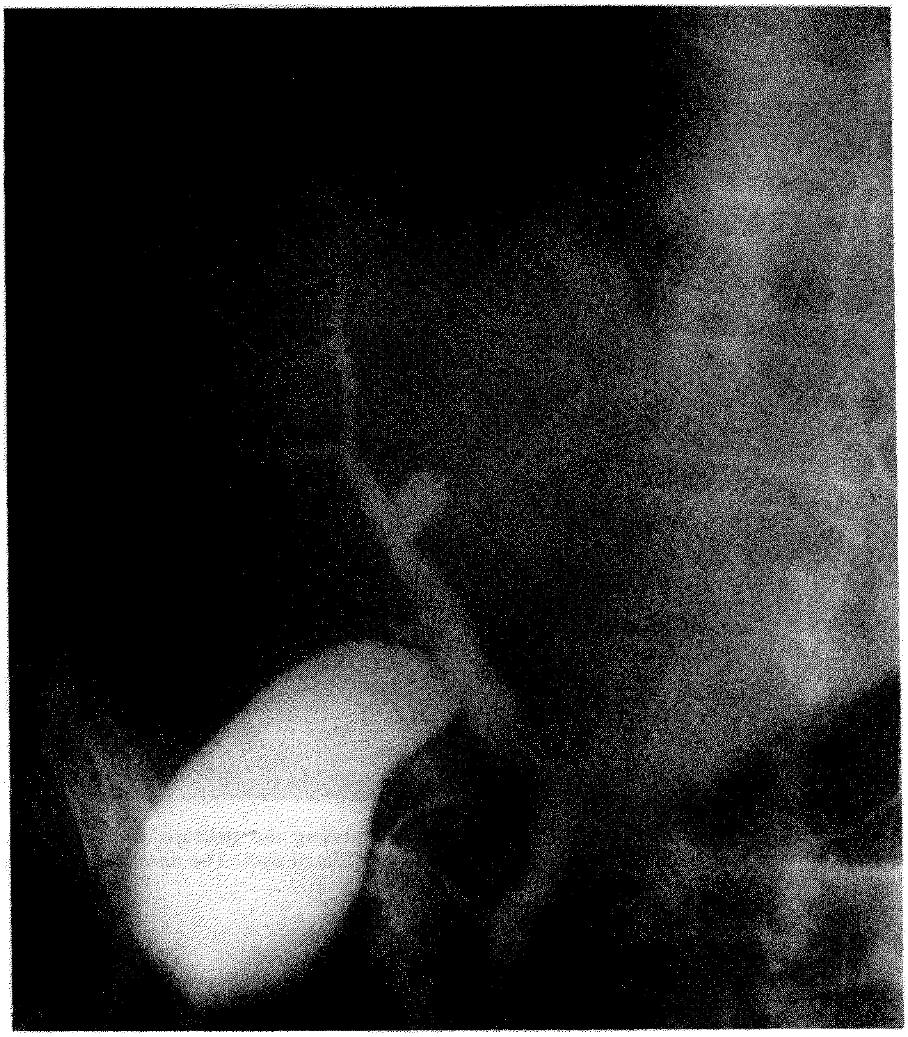

**Figure 1** Endoscopic retrograde cholangiogram via a nasobiliary catheter demonstrating the common bile duct, cystic duct, gall bladder, common hepatic duct, right anterior segmental hepatic duct and the origin of the left hepatic duct. Just above the cystic duct there is opacification of a small duct that is truncated. This is the obstructed right posterior segmental hepatic duct.

Cholangitis without jaundice has previously been reported due to a segmental duct obstruction[4]. The presence or absence of jaundice in this situation may depend on the functioning mass of liver drained by the obstructed duct[5]. Segmental duct obstruction should be suspected if ultrasound or CT scans demonstrate a focal area of duct dilatation within the liver. In the present case the ultrasound findings of dilated intrahepatic ducts associated with a stone, thought to be within the cystic duct but actually within the closely associated anomalous RPSHD, was suggestive of the Mirizzi syndrome[6]. This occurs when a calculus becomes impacted in the gallbladder neck or cystic duct and results in common hepatic duct stricturing by

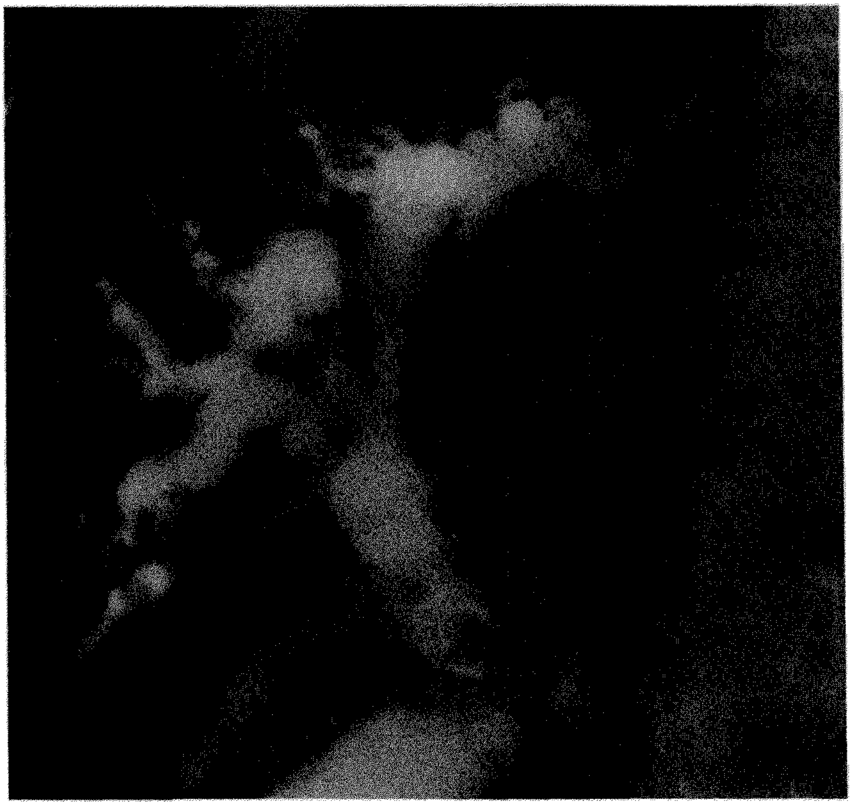

**Figure 2** Percutaneous transhepatic cholangiogram demonstrating the stricture in the distal right posterior segmental hepatic duct with multiple stones in the dilated duct. The nasobiliary catheter is seen within the common bile duct.

direct mechanical compression or associated inflammation. A calculus within an anomalous bile duct should therefore be excluded when this syndrome is being considered. The ERCP which was subsequently carried out failed to show any bile ducts in the right postero-lateral segment of the liver. This has been described as the "naked segment sign"[7] and required percutaneous cholangiography to define the anomaly.

A vascular ring obstructing the mid common hepatic duct has been documented as a rare cause of benign biliary stricture[8], but a vascular anomaly has not previously been reported as a cause of segmental hepatic duct obstruction. In the present case it would appear that the compression from the cystic artery branch was sufficient to eventually produce a fibrous stricture, possibly via bile stasis, infection and stone formation.

In a recent Japanese study 273 cases of hepatolithiasis were reviewed, 13 (4.8%) of which were due to congenital biliary tract abnormalities[9]. Caroli's disease was a common cause (9 cases), but none had anomalous extrahepatic bile ducts. In all

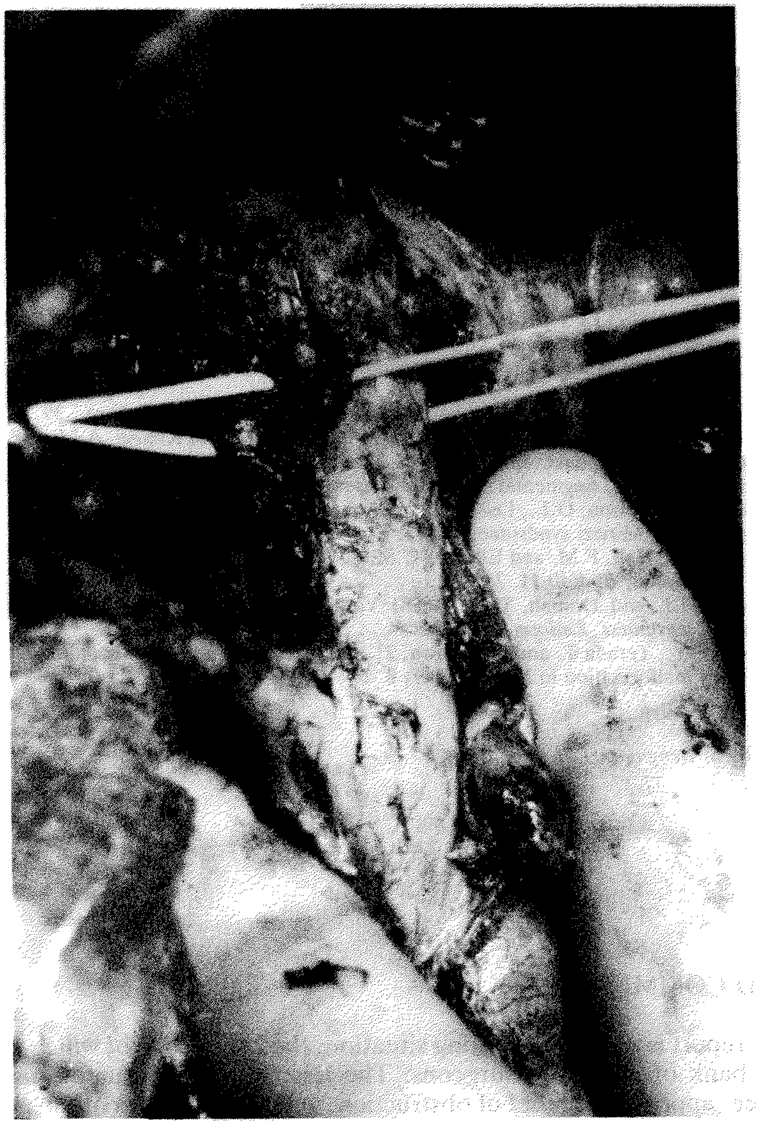

**Figure 3** Operative photograph demonstrating the common bile duct, common hepatic duct (yellow band) and the low insertion of the right posterior segmental hepatic duct (white band) into the common bile duct. (*See colour plate at back of issue*)

cases the stones were of brown pigment, as in the present case, suggesting that focal bile stasis as well as bacterial infection may have been responsible for their formation.

In summary, this case highlights the importance of an awareness of biliary tract abnormalities and the necessity for adequate evaluation of the biliary tree when investigations give conflicting results, and for appropriate treatment of hepatolithiasis associated with anomalous anatomy.

*Acknowledgements*

I should like to acknowledge the help and advice of the following people who have been involved in the management of this patient. Dr L Berger and Dr R Dick from the Department of Radiology and Dr O Epstein from the Department of Medicine, Royal Free Hospital, London and Mr J A McKinna from the Department of Surgery, Royal Marsden Hospital, Fulham Road, London.

*References*

1. Couinaud, C. (1957) Le Foie. Etudes anatomiques et chirurgicales. Volume 1: Paris: Masson
2. Healey, J.E. and Schroy, P.C. (1953) Anatomy of the biliary ducts within the human liver. Analysis of the prevailing patterns of branching and the major variations of the biliary ducts. *Arch. Surg.*, **66**, 599–616
3. Kune, G.A. (1969) The anatomical basis of liver surgery. *Aust. NZ J. Surg.*, **39**, 117–126
4. Braasch, J.W., Whitcomb, F.F., Watkins, E., Maguire, R.R. and Khazei, A.M. (1972) Segmental obstruction of the bile duct. *Surg. Gynecol. Obstet.* **134**, 915–20
5. Braasch, J.W. (1968) Segmental surgical disease of the liver. *Ann. Surg.*, **168**, 110–5
6. Koehler, R.E., Melson, G.L., Lee, J.K. and Long, J. (1979) Common hepatic duct obstruction by cystic duct stone Mirizzi syndrome. *Am. J. Roent.* **132**, 1007–1009
7. Sussman, S.K., Hall, F.M. and Elboim, C.M. (1986) Radiographic assessment of anomalous bile ducts. *Gastrointest. Radiol.* **11**, 269–72
8. Goldberg, H.J. and Doman, D.B. (1988) Vascular ring—an unusual cause of benign biliary stricture. *Gastrointest. Endosc.* **34**, 347–9
9. Nakanuma, Y., Terada T. and Nagakawa, T. *et al.* (1988) Pathology of hepatolithiasis associated with biliary malformation in Japan. *Liver*, **8**, 287–92

*(Accepted by S. Bengmark 17th June 1990)*

### INVITED COMMENTARY

This case report is a most interesting situation, the knowledge of which should be in the data bank of all biliary surgeons. The lessons are: adequate imaging of the biliary tree, appropriate relief of obstruction, and an awareness of the possibility of segmental or even lobar biliary obstruction with cholangitis but without jaundice. Obviously surgeons should read their own cholangiograms since it is not common knowledge that segmental obstruction can cause cholangitis.

It is difficult to ascribe obstruction of tubular structures to overlying arteries. Whether this mechanism was operative in this case is unknown. Short strictures do occur in the biliary tract without cause as exemplified by the cases in this commentary.

Herewith presented are two cholangiograms showing obstructed segments of the right lobe of the liver associated with stone disease and cholangitis. In the first, dilatation was achieved through a T tube tract to relieve the obstruction. The second was recognized at the time of choledochostomy by a routine cholangiogram and corrected by operative dilatation of the obstruction and extraction of stones.

John W. Braasch
Lahey Clinic
Burlington
Mass. U.S.A.

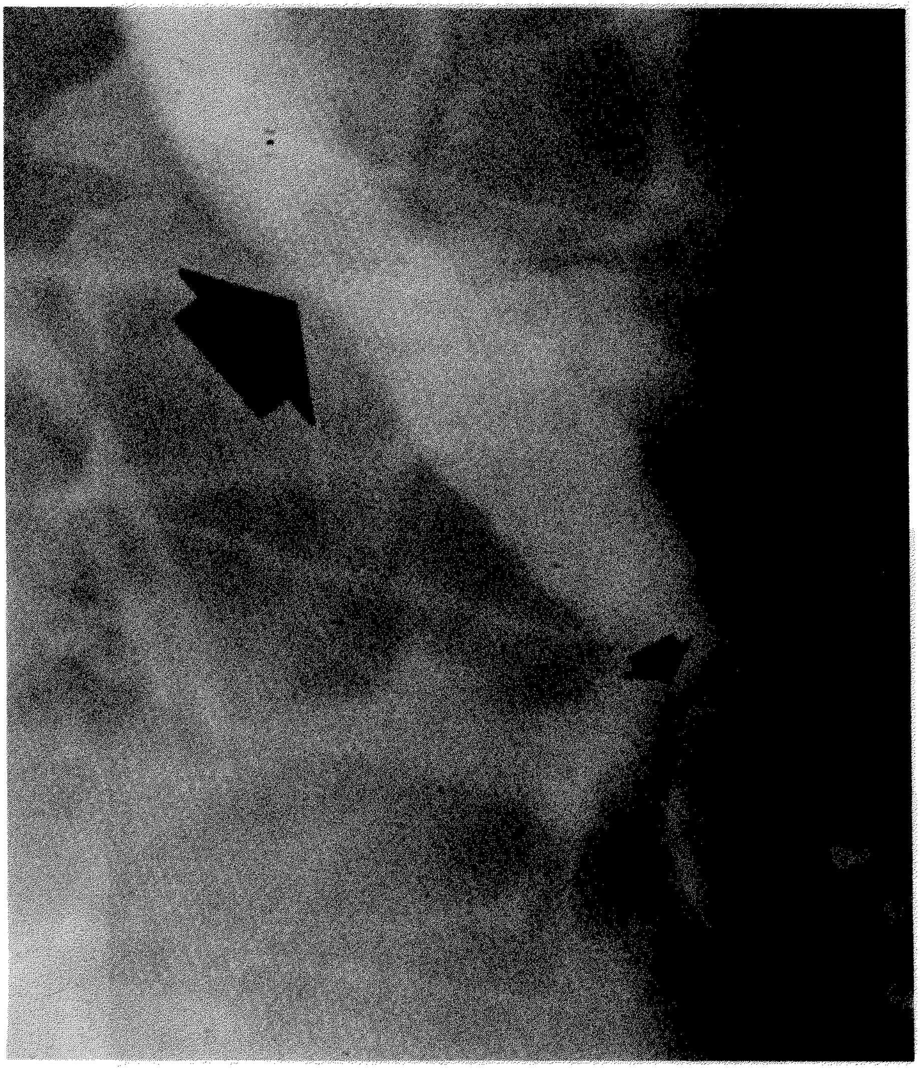

**Figure 1.** Transhepatic cholangiogram showing obstruction right segmental branch (small arrow) of the common duct (large arrow) with stones in obstructed segment (medium arrows).

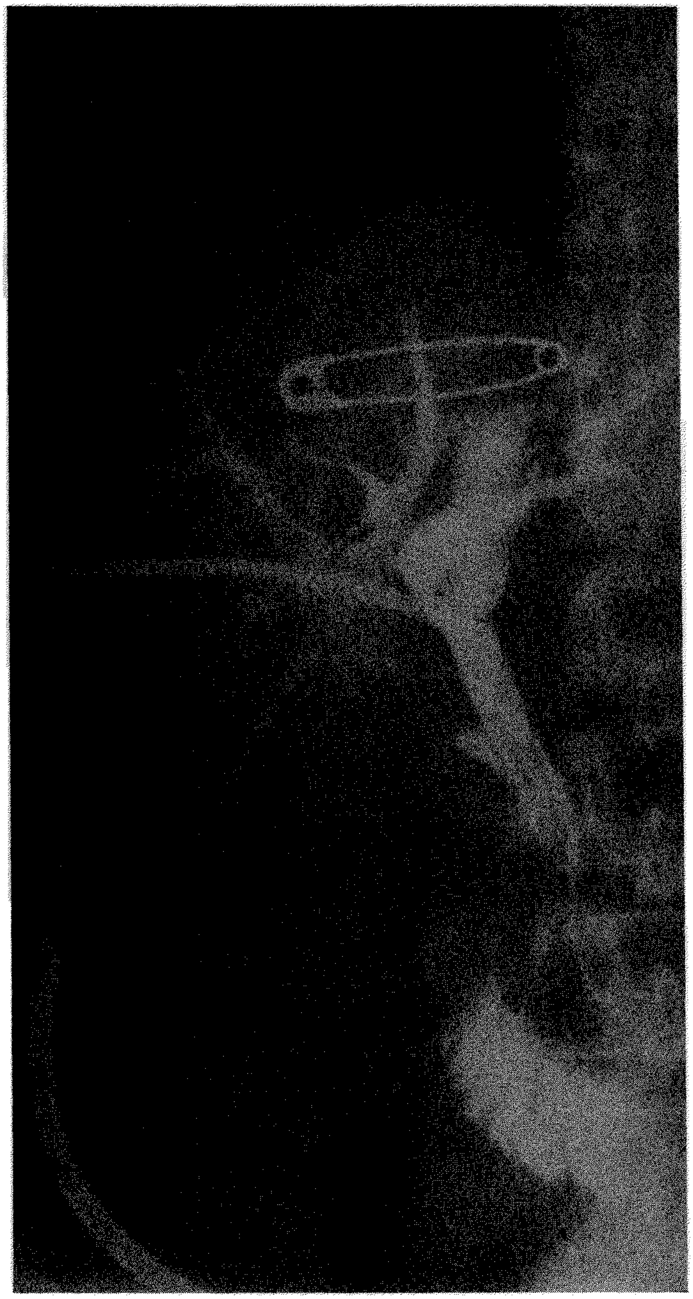

**Figure 2.** T-tube cholangiograms showing anomalous right hepatic duct with stones and obstructed distal junction with posterior aspect of common duct.

### INVITED COMMENTARY

This is an interesting paper discussing the pathogenesis and difficulties of treatment of intra-hepatic biliary stones.

As to the diagnosis of intra-hepatic stones, I support the view of the author on the importance of pre-operative investigation of the anatomy of the biliary tract, because the information on the whole architecture is indispensable in making a therapeutic plan. However, it is frequently difficult to obtain adequate x-rays because it is not easy to get contrast to enter the duct blocked by the stricture or involved in the anomalous anatomy. Abnormal drainage of the right posterior segmental duct into the common hepatic duct was noted in 8.8% of the patients with intra-hepatic stones in my personal series. This is a high incidence compared with that of normal subjects. In our Department, therefore, percutaneous trans-hepatic cholangioscopy with selective cholangiography is used to delineate the whole topography of the lesion in order to decide on the most suitable therapeutic procedure[1].

Regarding the pathogenesis of intra-hepatic stones in this particular case, the authors postulate that the stricture produced by a vascular ring which occurred incidentally was responsible for the formation of stones. This is a possible explanation in this case, but I am of the opinion that bile stasis and infection in the abnormal ducts with dilatation and then stricture formation produced by enteric organisms, which may arrive through the portal system, is a more likely cause for the stricture. I think that existence of the vascular ring is incidental and not the cause of the stricture. I presume that the inflammatory process occurred at the distal end of the aberrant duct where the stricture was found and involved the cystic duct which happened to pass there.

As to the treatment of intra-hepatic stones, the most satisfactory results are theoretically obtainable by release of strictures and complete removal of stones in those cases where hepatic lobectomy is not indicated[2]. In this particular case, a giant stone is impacted and must have migrated from the intra-hepatic ducts to the distal end of the right posterior segmental duct. There are in addition multiple stones in the dilated Segment VI and VII ducts, which are demonstrated on the cholangiogram. Moreover, less severe strictures, are also noted at the distal end of individual ducts. Therefore, complete removal of spontaneous migration of these stones would not be expected with the procedures described in this paper. Ascending cholangitis which would be more likely to occur in the environment produced by a bilioenteric anastomosis may contribute to the growth of pre-existing stones and the formation of new stones. In my opinion, segementectomy of Segment VI and VII would be the most adequate treatment in this particular case. If hepatic lobectomy was not indicated for some reason, complete removal of residual stones must be ensured with post-operative cholangioscopy.

*References:*

1. Yamakawa, T. (1989) How can cholangioscopy improve the management of intrahepatic stones. *Surg. Endosc.* **2**, 162–166
2. Wong, J. and Choi, T.K. (1984) Recurrent pygoenic cholangitis. In: Okuda, K., Nakayama F. and Wong, J. (eds). Intrahepatic calculi. Liss: New York, pp. 175–192

Tatsuo Yamakawa
Department of Surgery,
Teikyo University Hospital at Mizonokuchi,
Kawasaki, Japan

